# The Safety of Incisional Hernia Surgical Repair in Patients ≥70 Years

**DOI:** 10.7759/cureus.58322

**Published:** 2024-04-15

**Authors:** Islam Omar, Amr Elanany, Mohamed Ismaiel, Abby Townsend, Jeremy Wilson, Conor Magee

**Affiliations:** 1 General Surgery, The Hillingdon Hospitals NHS Foundation Trust, Uxbridge, GBR; 2 General Surgery, Charing Cross Hospital, Imperial College NHS Trust, London, GBR; 3 General Surgery, Altnagelvin Hospital, Londonderry, GBR; 4 General Surgery, Wirral University Teaching Hospital NHS Foundation Trust, Wirral, GBR

**Keywords:** outcomes, safety, old age, ventral hernia, geriatric, incisional hernia

## Abstract

Introduction

Incisional hernias (IHs) are common complications of abdominal surgery. Frailty and advancing age could be related to poor outcomes after surgical interventions, especially after operating on challenging surgical fields with adhesions and dense scars. This study assesses the safety of IH surgical repair in patients aged 70 years and above.

Methods

A retrospective analysis of all patients who had IH surgical repair on an emergency and elective basis at a district hospital in the UK. The cohort was categorised into group I (<70 years) and group II (≥70 years). A comparative analysis was conducted between these groups based on demographic data, comorbidities, hernia characteristics, operative data, and patient outcomes.

Results

This study encompassed 262 patients, with a mean age of 61.8 SD± 14.2 years, of whom 152 (58%) were females. Of these, group I comprised 173, and group II included 89 patients. Notably, group I exhibited a higher prevalence of morbid obesity, with 46 (28.8%) cases, as opposed to 12 (15.2%) in group II; p=0.021. Conversely, group II demonstrated a greater incidence of individuals with at least one comorbidity and chronic obstructive pulmonary disease (COPD) than group I, p=0.004 and 0.003, respectively. Fifty-five (32%) and 49 (29.3%) of group I had multiple defects and recurrent hernias compared to 24 (28.2%) and 16 (18.8%) in group II, p=0.541 and 0.071, respectively. The mean hospital stays were 5.5 ± 8.3 and 8.33 ± 18.7 days, and the mean durations of surgery were 131.6 ± 105.2 and 106.73 ± 74.22 minutes in groups I and II, p=0.057 and 0.181, respectively. No significant differences were observed in overall or wound-related complications, p=0.587 and 0.125. The rates of mortality within 30 days were three (1.7%) in group I and three (3.4%) in group II, with 90-day mortality rates at four (2.3%) and three (3.4%), respectively, indicating no significant difference. Similarly, no significant differences emerged between the groups regarding hernia recurrence rates (with a mean follow-up of 56 months) or 90-day readmission rates.

Conclusions

Surgical repair of IH is safe and effective in patients ≥70 years with comparable outcomes to younger patients.

## Introduction

Incisional hernias (IHs) are a common complication of open as well as minimal access abdominal surgery, with an estimated incidence of up to 30% [1,2]. Old age can lead to a vicious cycle in the context of IH [3]. First, advanced age is one of the recognized risk factors for IH development [4,5]. Additionally, IH in elderly patients is more likely to present with incarceration, necessitating emergency repair and potentially negative consequences [6-9]. However, although emergency surgery comes with specific risks, advanced age is also associated with negative postoperative outcomes in elective surgery [10].

Given the growing older population [11] and advances in anaesthesia techniques and perioperative care, many surgical candidates are in high-risk groups [12]. This could favour the adoption of a watchful wait policy with uncomplicated IH in older patients; however, such practice potentially increases the risk of presentation with incarceration and emergency surgery [1,13].

To address this common encounter, there should be an evidence-based discussion with patients before any surgical intervention. The aim of this work is to study the outcomes of surgical repair in patients ≥70 years old compared to their younger counterparts.

This article was previously presented as a meeting abstract at the 56th European Society for Surgical Research Congress (ESSR) on June 29, 2023.

## Materials and methods

Study design and setting

The present research comprises a comparative, retrospective cohort, single-centre study. The study was approved by the Trust's Ethics Committee (approval number 21_CA048). The patient data were obtained from a high-volume acute service district general hospital in the United Kingdom.

Participants

Any patients who had surgical repair of an IH and had complete data were considered eligible.

Variables examined

Data on the patient's demographics, comorbidities, and risk factors were collected. The primary outcome parameters included all-cause and wound-related complications and mortality rates. The secondary outcome parameters included length of hospital stay, need for high-level care, unplanned 90-day readmissions, and recurrence rate. The patients were categorised into two distinct cohorts: group I, <70 years old, and group II, ≥70 years old. The two groups were compared across the different variables examined.

Data source/bias

Data were collected from computerised records and patient case notes.

Study size

The total number of patients admitted for surgical repair of IHs with complete data.

Statistical analysis

The data were analysed using the SPSS software package version 20.0. (IBM Inc., Armonk, New York). The Kolmogorov-Smirnov test was used to verify the normality of data distributions. Continuous data were described using range (minimum and maximum), mean, standard deviation, and median. Comparisons between groups for categorical variables were assessed using the Chi-squared test (Fisher or Monte Carlo). A Student's t-test was used to compare normally distributed quantitative variables across the two groups. A Mann-Whitney test was used to compare non-parametric data. The results were deemed significant at the 5% threshold.

## Results

The study included 262 patients with a mean age of 61.8 SD ± 14.2 years; 152 patients (58%) were female. Groups I and II included 173 and 89 patients, respectively. A comparison of demographics and comorbidities (Table 1) indicated that group I had more patients with morbid obesity than group II (body mass index (BMI) ≥35), with 46 patients (28.8%) in group I being morbidly obese in comparison to 12 (15.2%) in group II; p=0.021. Furthermore, group II exhibited a greater prevalence of patients with at least one comorbidity and COPD compared to group I, p=0.004 and 0.003, respectively. Additionally, 27 patients (15.7%) in group I had malignancy, in comparison to 28 (31.5%) in group II; p=0.003.

**Table 1:** Relation between age and demographics, comorbidities, and other risk factors (n=262) FE - Fisher exact; U - Mann-Whitney test; t - student's t test *: Statistically significant at p ≤ 0.05

Variables	Age (years)		Test of sig.	p-value
	<70	≥70		
Gender	(n = 173)	(n = 89)		
Male	73 (42.2%)	37 (41.6%)	x^2^=0.009	p=0.923
Female	100 (57.8%)	52 (58.4%)		
BMI (kg/m^2^)	(n = 160)	(n = 79)		
<35	114 (71.3%)	67 (84.8%)	x^2^=5.291^*^	p=0.021^*^
≥ 35	46 (28.8%)	12 (15.2%)		
Any comorbidity	(n = 173)	(n = 89)		
No	83 (48%)	26 (29.2%)	x^2^=8.516^*^	p=0.004^*^
Yes	90 (52%)	63 (70.8%)		
DM	(n = 173)	(n = 89)		
No	147 (85%)	69 (77.5%)	x^2^=2.249	p= 0.134
Yes	26 (15%)	20 (22.5%)		
Malignancy	(n = 172)	(n = 89)		
No	145 (84.3%)	61 (68.5%)	x^2^=8.762^*^	p=0.003^*^
Yes	27 (15.7%)	28 (31.5%)		
Connective tissue disease	(n = 173)	(n = 89)		
No	166 (96%)	86 (96.6%)	x^2^=0.073	^FE^p=1.000
Yes	7 (4%)	3 (3.4%)		
COPD	(n = 173)	(n = 89)		
No	162 (93.6%)	73 (82%)	x^2^=8.583^*^	p=0.003^*^
Yes	11 (6.4%)	16 (18%)		
CKD	(n = 172)	(n = 89)		
No	160 (93%)	82 (92.1%)	x^2^=0.069	p=0.793
Yes	12 (7%)	7 (7.9%)		
Other risk factors				
Chemotherapy	(n =173)	(n =89)	x^2^=2.167	^FE^p=0.192
No	168 (97.1%)	83 (93.3%)		
Yes	5 (2.9%)	6 (6.7%)		
Radiotherapy	(n =173)	(n =89)	x^2^=0.001	^FE^p=1.000
No	171 (98.8%)	88 (98.9%)		
Yes	2 (1.2%)	1 (1.1%)		

Comparison of the hernia characteristics (Table 2) revealed that 55 (32%) and 49 (29.3%) of group I had multiple defects and recurrent hernias compared to 24 (28.2%) and 16 (18.8%) in group II, p=0.541 and 0.071, respectively. A variety of surgical techniques were used according to patient and hernia characteristics, presentation, and surgeon preference. One hundred thirty-three (80.1%) from group I and 73 (85.9%) from group II had open repair, 31 (18.7%) from group I, and 12 (14.1%) from group II had laparoscopic repair. The mesh placement techniques used included sublay, onlay, inlay and intraperitoneal onlay mesh repair (IPOM). Additionally, we observed that vicryl meshes were used more often in the emergency setting as a bridge to the definitive abdominal wall reconstruction at a later stage. 

**Table 2:** Relation between age and hernia characteristics, operative details, and outcome parameters (n=262) FE - Fisher Exact; U - Mann-Whitney test; MC - Monte Carlo; #: Continuous data were described using range (minimum and maximum), mean, standard deviation, and median *: Statistically significant at p ≤0.05

Variables	Age (years)		Test of sig.	p
	<70	≥70		
Stoma on presentation	(n =168)	(n =84)		
No	157 (93.5%)	78 (92.9%)	x^2^=0.032	p=0.859
Yes	11 (6.5%)	6 (7.1%)		
Multiple defects	(n = 172)	(n = 85)		
No	117 (68%)	61 (71.8%)	x^2^=0.374	p=0.541
Yes	55 (32%)	24 (28.2%)		
Primary/Recurrent	(n = 167)	(n = 85)		
Primary	118 (70.7%)	69 (81.2%)	x^2^=3.256	p=0.071
Recurrent	49 (29.3%)	16 (18.8%)		
Mode	(n =166)	(n =85)		
Lap	31 (18.7%)	12 (14.1%)	x^2^=1.479	^MC^p=0.417
Open	133 (80.1%)	73 (85.9%)		
Assisted	2 (1.2%)	0 (0.0%)		
Adhesiolysis	(n = 168)	(n = 83)		
No	89 (53%)	39 (47%)	x^2^=0.797	p=0.372
Yes	79 (47%)	44 (53%)		
Duration of surgery (min.) #	(n = 167)	(n = 82)		
Median (Min. – Max.)	103 (18 – 604)	88.5 (11 – 375)	U=6133.0	p=0.181
Mean ± SD.	131.6 ± 105.2	106.73 ± 74.22		
Postop HDU/ICU	(n =169)	(n =87)		
No	149 (88.2%)	72 (82.8%)	x^2^=1.423	p=0.233
Yes	20 (11.8%)	15 (17.2%)		
Hospital Stay (days) #	(n =173)	(n =89)		
Median (Min. – Max.)	3 (0 – 57)	5 (0 – 169)	U=6598.500	p=0.057
Mean ± SD.	5.5 ± 8.3	8.33 ± 18.7		
Clavien-Dindo	(n =173)	(n =89)		
No CD	93 (53.8%)	48 (53.9%)	x^2^=4.697	^MC^p=0.587
CDI	33 (19.1%)	15 (16.9%)		
CDII	25 (14.5%)	17 (19.1%)		
CDIIIA	8 (4.6%)	1 (1.1%)		
CDIIIB	9 (5.2%)	3 (3.4%)		
CD IV	2 (1.2%)	2 (2.2%)		
CDV	3 (1.7%)	3 (3.4%)		
Wound complications	(n =172)	(n =89)		
No	140 (81.4%)	79 (88.8%)	x^2^=2.359	p=0.125
Yes	32 (18.6%)	10 (11.2%)		
Mortality 30 days	(n =172)	(n =89)		
No	169 (98.3%)	86 (96.6%)	x^2^=0.691	^FE^p=0.413
Yes	3 (1.7%)	3 (3.4%)		
Mortality 90 days	(n =172)	(n =89)		
No	168 (97.7%)	86 (96.6%)	x^2^=0.245	^FE^p=0.693
Yes	4 (2.3%)	3 (3.4%)		
Recurrence	(n =172)	(n =89)		
No	154 (89.5%)	82 (92.1%)	x^2^=0.458	p=0.499
Yes	18 (10.5%)	7 (7.9%)		
90 days readmissions	(n =172)	(n =89)		
No	142 (82.6%)	78 (87.6%)	x^2^=1.144	p=0.285
Yes	30 (17.4%)	11 (12.4%)		

The comparison of the outcomes (Table 2) revealed no significant differences between the two groups. Specifically, there were no significant variations in overall or wound-related complications (p=0.587 and 0.125, respectively). In group I, 32 (18.6%) cases developed wound complications compared to 10 (11.2%) cases in group II. We had only one case of mesh infection in group I, which required mesh removal. The patient was a 34-year-old female with a high BMI (42 kg/m^2^) who presented with an incarcerated incisional hernia (10 x 10cm defect) and underwent emergency surgical repair. Mesh infection was treated initially with antibiotics. However, the onlay mesh was eventually removed to achieve complete resolution.

The surgical repair seemed challenging, with 79 (47%) cases of group I and 44 (53%) of group II requiring adhesiolysis. We encountered three iatrogenic enterotomies, two in group I <70 years and one in group II ≥70 years. All of them had adhesiolysis. One had laparoscopic IH repair, and two had open repair.

The mean hospital stays were 5.5 ± 8.3 and 8.33 ± 18.7 days in groups I and II, respectively, with a marginally more extended stay for group II, p=0.057. Twenty (11.8%) patients from group I and 15 (17.2%) from group II needed a high-level of care, high-dependency unit/intensive therapy unit (HDU/ITU); however, the difference between groups was not significant p=0.233.

The 30-day mortality rates were observed to be three patients (1.7%) in group I and three patients (3.4%) in group II. In comparison, the 90-day mortality rates were four patients (2.3%) in group I and three patients (3.4%) in group II, with no statistically significant differences found between the two groups. Additionally, there were no significant differences in either the recurrence rate over an average follow-up period of 56 months or the 90-day readmissions between both groups. 

## Discussion

This study demonstrated the safety of IH surgical repair in patients ≥70 years of age; older patients had comparable outcomes to their younger counterparts.

IH is one of the most common surgical presentations to general surgical teams and represents a challenge in terms of management options and costs [14]. A recent French publication [15] studied the patients who had abdominal surgery in 2013-2014. Out of 710,074 patients, 32,633 (4.6%) and 5117 (0.7%) had ≥1 and ≥2 incisional hernia repair(s) within five years of follow-up, respectively. The mean hospital costs were estimated at €4153 for each hernia repair, with overall costs of €67.7 million/year.

This study confirmed the prevalence of comorbidities in the older patient group; more than 70% of the patients aged ≥70 years had at least one comorbidity compared to 52% of group I. A more detailed analysis revealed that in group II, 18% had COPD and 31.5% had malignancy, compared to 6.4% and 15.7%, respectively, in group I. These findings could reflect, in part, the challenge and risk of surgical interventions in the older patient group. However, our data revealed a higher incidence of morbid obesity in the younger patient group, with 28.8% having a BMI ≥35 kg/m2 compared to 15.2% in the older patient group.

We studied the hernia characteristics of relevance. In the full cohort, 25.8% had recurrent hernias, 30.7% had multiple defects, and 6.7% had a stoma on presentation. These figures indicate a cohort with complex IH, and this has been reflected in surgical management, where 49% of the cases needed adhesiolysis and 82.1% had open repair with a mean duration of surgery of 123.4 ± 96.7 minutes and a median duration of 95 minutes (IQR 58-170 minutes). Despite that, our outcomes were comparable to those of similar studies [10,16]. In this study, 16.1% of patients had wound complications, and the 30-day and 90-day mortality rates were 2.3% and 2.7%, respectively. The recurrence rate was 9.6%, and the 90-day unplanned readmissions rate was 15.7%.

Comparing the outcomes between the two groups revealed no significant differences in terms of overall and wound-related complications, mortality rates, readmissions, and recurrence. A recent study [17] of the Herniamed Registry included 46,040 patients who had IH repair. They compared the outcomes of patients 80 years of age and older to those younger than 80 years. They found higher complication rates in the older versus younger patient group (5.5% versus 3.0%, respectively; p<0.001). Reoperation rates were also higher with increased age (3.8% for older versus 3.0% for younger patients; p=0.007). However, the recurrence rates and pain were lower in the older patient group. They concluded that IH repair in patients 80 years of age and older is associated with a slightly higher complication risk but is acceptable and also has improved pain scores.

Our data indicated an overall acceptable outcome for surgical repair of IH in patients ≥70 years of age. These findings were echoed in a study that included 136 patients who underwent complex abdominal wall hernia repair with porcine-derived biologic mesh [18]. They categorized the patients into two groups: elderly (≥65 years) and nonelderly (18-64 years), and examined several outcomes comprising surgical site infection, mortality, reoperation rate, and requirement for mechanical ventilation and hospital stay via propensity-matched analysis. Furthermore, they revealed no significant difference between the two groups. Hence, age should not be considered a contraindication for surgical repair. 

Generalizability

This study has been conducted in a high-volume acute service facility in the UK for over seven years. The cohort included is representative of the population served in most low- and middle-income countries. The results and conclusions from this study will be helpful for similar healthcare facilities.

Strengths

There was an extensive analysis of the potential confounding factors, including the comorbidity status and risk factors for adverse outcomes. The study has been formulated and presented according to the STROBE guidelines.

Limitations

The limitations of this study included the retrospective nature and relatively small sample size. Moreover, the fact that all data were from a single centre could potentially compromise generalizability.

## Conclusions

The surgical outcomes of IH repair in patients ≥70 years old are comparable to those of their younger counterparts without significant differences in complications and mortality. Our study demonstrated the overall safety of surgical repair of IH in patients ≥70 years old. Age should not be considered a contraindication to the definitive repair of IH.
